# Aquapuncture Using Stem Cell Therapy to Treat Mdx Mice

**DOI:** 10.1155/2015/132706

**Published:** 2015-05-13

**Authors:** Greyson Vitor Zanatta Esper, Graciela Conceição Pignatari, Marcio Nogueira Rodrigues, Heloisa Godoi Bertagnon, Isabella Rodrigues Fernandes, Nanci Nascimento, Angela Maria Florencio Tabosa, Patrícia Cristina Baleeiro Beltrão-Braga, Maria Angelica Miglino

**Affiliations:** ^1^Stem Cell Lab, Surgery Department, School of Veterinary Medicine, University of São Paulo, 87 Prof. Dr. Orlando Marques de Paiva Avenue, 05508-270 São Paulo, SP, Brazil; ^2^Center for Cellular and Molecular Studies and Therapy (NETCEM), São Paulo, SP, Brazil; ^3^Clinic Department, School of Veterinary Medicine, University of São Paulo, 87 Prof. Dr. Orlando Marques de Paiva Avenue, 05508-270 São Paulo, SP, Brazil; ^4^Nuclear and Energy Research Institute Biotechnology Center, 2242 Prof. Lineu Prestes Avenue, 05508-270 São Paulo, SP, Brazil; ^5^Division of Chinese Medicine and Acupuncture, Federal University of São Paulo, 862 Botucatu Street, 04023-900 São Paulo, SP, Brazil; ^6^Obstetrics Department, School of Arts, Sciences and Humanities, University of São Paulo, 1000 Arlindo Bettio Avenue, 03828-000 São Paulo, SP, Brazil

## Abstract

Duchenne muscular dystrophy (DMD) occurs due to genetic mutations that lead to absence or decrease of dystrophin protein generating progressive muscle degeneration. Cell therapy using mesenchymal stem cell (MSC) has been described as a treatment to DMD. In this work, MSC derived from deciduous teeth, called stem cells from human exfoliated deciduous teeth (SHED), were injected in acupoint as an alternative therapy to minimize muscle degeneration in twenty-two mdx mice. The treatment occurred three times with intervals of 21 days, and animals were analyzed four times: seven days prior treatment (T-7); 10 days after first treatment (T10); 10 days after second treatment (T31); and 10 days after third treatment (T52). Animals were evaluated by wire test for estimate strength and blood was collected to perform a creatinine phosphokinase analysis. After euthanasia, cranial tibial muscles were collected and submitted to histological and immunohistochemistry analyses. Treated groups presented improvement of strength and reduced creatinine phosphokinase levels. Also, a slight dystrophin increase was observed in tibial cranial muscle when aquapuncture was associated SHED. All therapies have minimized muscle degeneration, but the association of aquapuncture with SHED appears to have better effect, reducing muscle damage, suggesting a therapeutic value.

## 1. Introduction

Muscular dystrophies are neuromuscular diseases genetically related that compromise progressively skeletal muscles. Regarding that, Duchenne muscular dystrophy (DMD) presents worldwide frequency of approximately 1 : 3500 male children. DMD is a recessive inheritance pattern, with the first symptoms starting at 3–6 years old [1–3]. The DMD mutation occurs in a gene located on the X chromosome (Xp21), responsible for encoding dystrophin [1, 4], a structural protein that acts stabilizing the sarcolemma muscle, connecting the actin cytoskeleton with the extracellular matrix [5]. Dystrophin deficiency affects the contraction physiology, causes sarcolemma injury, and leads to cell death [6].

Several studies tried some therapeutic strategies to treat DMD, such as antibiotics, glucocorticoids, and calcium channel blockers [7–11]. Cell therapy using stem cells has also been tested [9, 11–15]. Besides stem cell hypothetically could replace damaged cell, and stem cells also are able to perform immunomodulation activities that could balance the progression of muscular dystrophy by anti-inflammatory effect [7, 14, 16–18]. In addition, gene therapy has been tested to treat DMD [19–22] and, recently, a combination of stem cells, using induced pluripotent stem cells (iPSC) from a patient with DMD, and gene therapy, correcting genetically the cells using TALENs or CRISPR, restored dystrophin production* in vitro* [22].

Stem cells from human exfoliated deciduous teeth (SHED) were first isolated as a population of multipotent stem cells from the pulp of exfoliated deciduous teeth [23]. SHED are able to differentiate into odontogenic, adipogenic, osteogenic, neural, hepatocytes, and endothelial cells [24–27], have immunomodulatory properties [27, 28], and are able to perform cell reprogramming [29]. Also, stem cells from dental pulp were used to treat Golden Retriever muscular dystrophy dogs providing some dystrophin expression [30].

The dystrophin deficient mice, mdx (C57BL10 ScSn-DMD-mdx/J) are widely used as an animal model to study DMD in order to investigate various aspects of this disease and for preclinical test or proof-of-concept studies. Mdx mice have a milder clinical behavior than human, presenting muscle degeneration in waves leading to necrosis, as well as presenting some serum biochemical parameters and histological changes, similar to human affected by DMD [31, 32].

Acupuncture, a traditional oriental medicine is therapeutic approach that has been used to contribute to pain control, analgesia, inflammatory process, and others factors improving the treatment in several degenerative diseases like Parkinson's, DMD, and spinal cord injury [33–36]. Aquapuncture is a variation of acupuncture that involves the application of aqueous substances (like drugs) in the acupoints in order to potentiate therapeutic action [37]. This technique combines acupoints stimulation and the pharmacological substances improving beneficial effect if used separately [37]. The association of acupuncture with cell therapy is new, but some studies in this direction have already been performed. Recently, acupoints injection associated with mesenchymal stem cell (MSC) was used to treat hip dysplasia in dogs presenting positive results, showing increased functional improvement [38].

Regarding this possible beneficial effect, this study aimed to use acupoint injection with SHED in order to verify the benefits of this approach to treat mdx mice.

## 2. Materials and Methods

### 2.1. Animals

This work was approved by the ethics committee on animal use, from the School of Veterinary Medicine and Animal Science, São Paulo University, protocol number 2431/2011. Animal research was conducted in accordance with the Ethical Principles in Animal Experimentation, according to the National Institute of Health Guide for the Care and Use of Laboratory Animals, 1996. Twenty-two male mdx mice (14–20 g) between 4 and 6 weeks of age from IPEN animal facility (Nuclear and Energy Research Institute, Brazil) were used in this work. Animals were kept in cage (40 × 34 × 17 cm) with wood shavings, light/dark cycle (12/12 h) in controlled temperature (20 ± 1°C) and humidity (50 ± 10%), feed (Nuvital, PR, Brazil), and water ad libitum. The maximum number of animals per cage was 5, divided according with treatment received.

### 2.2. SHED

SHED were obtained using a modified Miura's protocol [12], with collagenase type I for pulp digestion (3 mg/mL). SHED were carefully prepared to animal infusion. After trypsinization, cells were washed twice and counted and 1 × 10^4^ cells were ressuspended in saline solution (Sigma-Aldrich, MO, USA) in a very low volume (20 microliters).

### 2.3. Acupoint Associated with Stem Cell Injection

**Figure 1:**
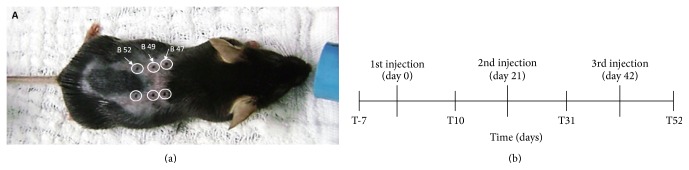
Acupoint injections and timeline of treatments. (a) Acupoints used in this study in mdx mice: BL47, BL49, and BL52 (white arrows). (b) Experimental timeline considering days of injection and days for treatment evaluation. (T-7) seven days before treatment; (T10) ten days after first treatment (SHED or saline injection); (T31) twenty-one days after second treatment (SHED or saline injection); (T52) twenty-one days after third treatment (SHED or saline injection).

Four groups of animals were distributed to do the following treatments: (A) SHED injection in false acupoint (*n* = 5); (B) saline injection in true acupoint (*n* = 5); (C) SHED in true acupoint (*n* = 5); and (D) control, without treatment (*n* = 5). SHED were injected in each acupoint every three weeks (21 days of interval each), totaling three injections during the whole experiment. Acupoints selected for injection were Bladder 47 (Hunmen), Bladder 49 (Yishe), and Bladder 52 (Zhishi) (Figure 1(a)), selected based on Traditional Chinese Medicine because these acupoints can connect the meridian and could improve the effect in the body. After treatments mdx were analyzed at four moments: (T-7) seven days before treatment; (T10) 10 days after the first SHED transplantation; (T31) 10 days after the second SHED transplantation; and (T52) 10 days after the third SHED cell transplantation, according to the timeline (Figure 1(b)). Before the injection mice used chemical restraint and inhalational anesthesia in an open system with isoflurane 3% (Cristália, SP, Brazil). Placebo groups were injected with 20 microliters of saline solution at same acupoints and conditions.

### 2.4. Acupoint Treatment Analyses

Animals were submitted to clinical evaluation by wire test, creatinine phosphokinase, and histological analyses.

#### 2.4.1. Wire Test

The strength of the animal's forelimbs was assessed by wire test adapted from van Putten et al. (2012) [6]. For this test, mice were suspended on a metal cloth hanger secured 35 cm above the cage. Animals were analyzed until the fall, for 4 consecutive times, all recorded by video. The forelimbs strength was quantified using a rating scale considering the following parameters: (1) raise the body above the wire, (2) walk on the wire, (3) put the pelvic member on the wire; and (4) time on the wire. Each parameter was analyzed by three double-blind raters (Table 1). After this evaluation the values obtained in Table 1 were summed for each animal and the animals were classified in poor, median, and strong according to muscle strength score. Results were plotted in GraphPad Prism 5 and analyzed using 2-way ANOVA.

#### 2.4.2. Analysis of Serum Creatinine Phosphokinase (CPK)

Animals' blood was collected in the facial vein and plasma was diluted 1 : 100. The samples were analyzed kinetically by CK-NAC kit (Randox Laboratories, London, England) in Biotek Powerwave XS spectrophotometer by the KC4 software (v. 3.4) (Biotek, CA, USA). Results were plotted in GraphPad Prism 5 and analyzed using 2-way ANOVA.

#### 2.4.3. Histological Analysis

After animal's euthanasia, cranial tibial muscle and diaphragm were collected, frozen in liquid nitrogen, fixed in 4% paraformaldehyde, and subjected to routine histological procedures. Sections of 5 *μ*m thickness were performed in a microtome (Leica RM-2065, Germany) and stained with hematoxylin-eosin (HE) and visualized by light microscopy (Olympus BX60 microscope, Tokyo, Japan) coupled with an AxioCam (Zeiss, Jena, Germany). Immunofluorescence analyses on tibial cranial and diaphragm muscles sections were performed to analyze the presence of dystrophin, using serial sections of 10 *μ*m thickness accommodated on silanized slides. C57/BL6, animal without dystrophin pathology, was used here as a positive control for dystrophin expression. The samples were fixed with 100% acetone for 20 minutes at −20°C. Then, the slides were washed with PBS 3 times for 5 min each and added anti-dystrophin antibody (ab3149, Abcam, Cambridge, UK) diluted (1 : 100) in PBS containing 1% bovine serum albumin (BSA) in a humid chamber overnight at 4°C. After this time, the samples were washed with PBS 3 times, 5 min each, and added secondary anti-rabbit IgG NL557 antibody (R&D Systems, Minneapolis, USA) diluted 1 : 200 in PBS containing 1% BSA, kept in a dark chamber for 60 minutes at room temperature. Then, the slides were washed with PBS 5 times for 5 minutes each and mounted using Vectashield with DAPI (Vector Laboratories, Burlingame, CA, USA).

## 3. Results and Discussion

Here we proposed an alternative method to treat DMD using mdx as a model to test the benefits of acupuncture associated with stem cell therapy, a pioneer work in this field. Acupoints were selected bilaterally after an accurate assessment of DMD, associated with the benefits they could bring from Traditional Chinese Medicine. So, in this project the points used to apply acupuncture were Bladder 47 (B47), Bladder 49 (B49), and Bladder 52 (B52) based on the fact that DMD patients present a deficiency in liver, spleen-pancreas, and kidney meridians and the points chosen could nourish the first column of Shu points [39]. The B47 point is located on the side depression to the edge flow, related to the spinous process of the vertebra. The B49 point is located on the side depression of the spinous process of the twelfth vertebra and is related to the spinous process of the vertebra. The B52 point is located on the side depression of the spinous process of the second lumbar vertebra, which is also related to the spinous processes of the vertebrae (Figure 1(a)).

In this work, mdx mice were evaluated for the total time of 52 days (Figure 1(b)), which is considered enough time to check mdx muscle degeneration according to natural disease evolution in this animal model [40]. As here mdx mice remained less time on the wire and fatigue more than observed by van Putten's wire test [6]. For this reason the test was adapted including other parameters, such as erection of the body on the wire, walking on the wire, and placement of the pelvic limb on the wire. In addition, scales for scoring behaviors and the quality of strength, like weak, medium, and strong, were assigned (Table 1).

**Figure 2:**
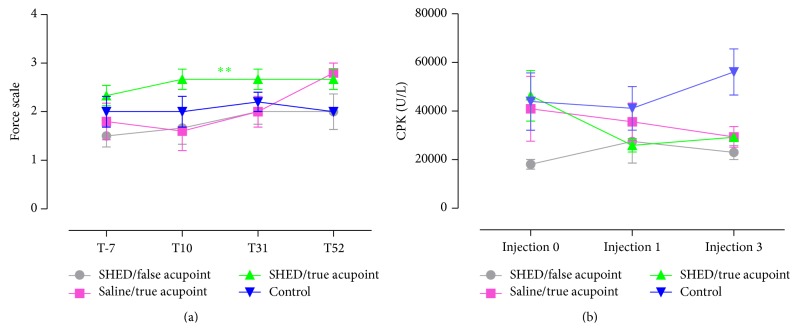
Analysis for the treatment using wire test and seric CPK, (a) scale force graphic using wire test, (b) seric CPK analysis. ∗∗  means *p* value = 0.0021.

Concerning force using an adapted wire test, statistical differences were observed comparing treatments, with a strength improvement in animals submitted to SHED/true acupoints (*p* value: 0.0021) and a slight strength improvement in SHED/false acupoints and saline/true acupoints, always comparing with animal without any treatment, called controls (Figure 2(a)). However, the improvement of force in SHED true acupoint suggested clinical progress in muscle which is interesting making acupuncture an alternative therapy but not enough to revert the physiological behavior. Force improvement using just acupuncture was related before using the E36, BP6, and auricular Shenmen points in mdx showing a positive result [41], like observed here. Moreover, concerning stem cell treatment to mdx, when adipose stem cells were injected intramuscularly in mdx mice increasing muscle strength and resistance to muscle fatigue were observed [39].

Serum CPK analysis is a reliable measurement for the identification and quantification of muscle damage. This analysis infers animals' improvement or worsening during the treatment [42–44]. Increasing of CPK suggests muscle damage during the peak of muscle degeneration in mdx mice, which occurs between 35 and 90 days of age [40]. Here, CPK decreased approximately 1.5 times less than the control using SHED/true acupoints, which suggest a clinical improvement in this animal group. Seric CPK revealed that all treated animals presented CPK decrease, although significant differences were not found (Figure 2(b)).

The causes of the perpetuation of muscle degeneration are numerous such as free radicals release and lack of the dystrophin [45, 46]. Therefore, stem cells effect in false acupoints or placebos could result in the same mechanism of action, explaining the reduction in plasma CPK among treatments. The CPK decreasing is a good parameter to monitor the treatment [47, 48].

In addition, we observed a delay in disease progression in animals treated with SHED in false acupoints suggesting that cells could present a paracrine effect, which is already, known for SHED [27].

**Figure 3:**
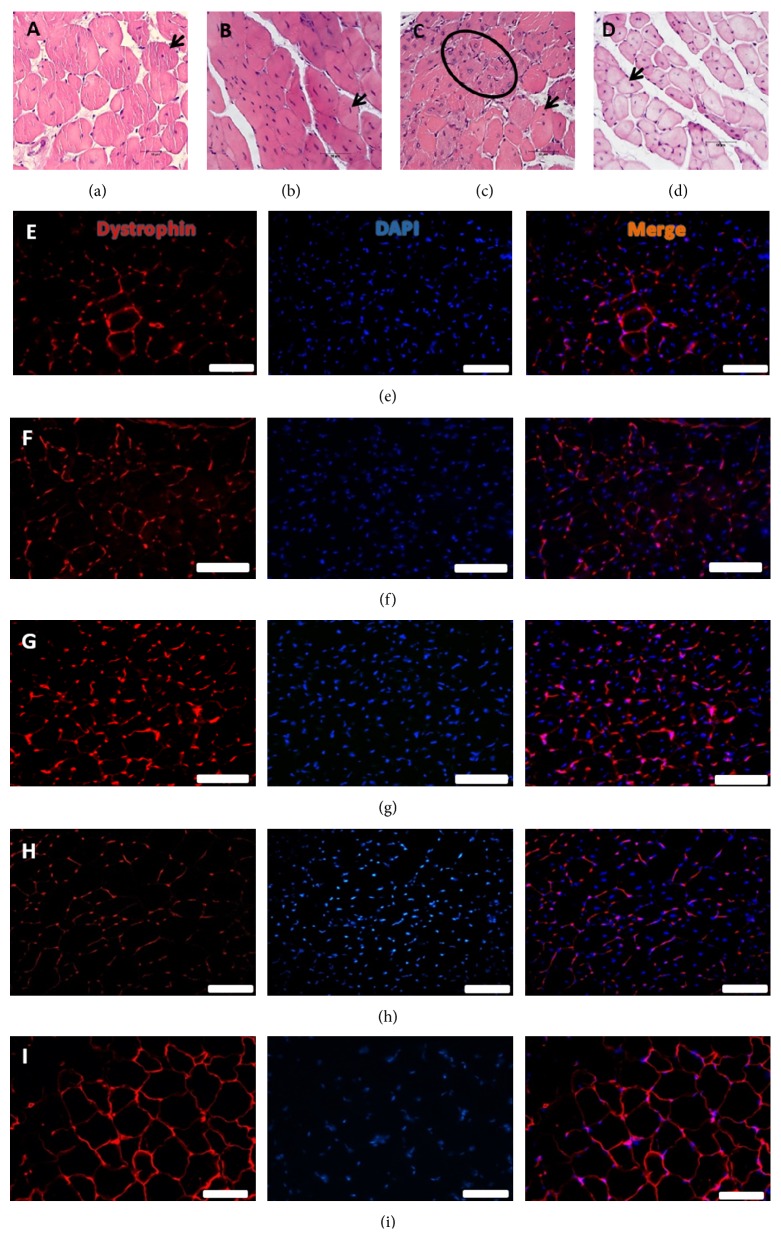
Histological analyses of cranial tibial muscle using HE staining (a–d). In (c), inside the circle, centralized nuclei and increased heterogeneity in myofibers size, characteristics of mdx muscle degeneration. Dystrophin expression in tibial cranial muscles after 52 days of the treatment (e–i). Groups: SHED/false acupoints; saline/true acupoints; SHED/true acupoints; and control. Scale Bar—100 *μ*m.

Histological analysis inferred that treatments did not interfere with anatomical structure of tissue and did not produce inflammatory reaction (Figures 3(a)–3(d)).

Immunohistochemistry using dystrophin protein analysis in cranial tibial muscle in mdx revealed that all treated groups presented a slight dystrophin improvement (Figures 3(e)–3(g)), comparing with the control (mdx without treatment Figure 3(h)), being more evident in SHED/true acupoint and SHED/false acupoint, suggesting cell influence. However, even getting a little better, visually, dystrophin amount was lower in mdx mice than in C57/BL6, animal without dystrophin pathology, used here as a positive control for dystrophin expression (Figure 3(i)). In spite of mdx mice treated with SHED in true acupoint presented an increase of dystrophin expression in tibial cranial muscle; this was not sufficient to improve muscle tissue normal appearance (Figure 3(g)), suggesting that regenerative ability might not be directly related to dystrophin presence. The increase of dystrophin production was also observed in mdx mice treated with some drugs, as gentamicin, pentoxifylline, and RTC 13 and 14 molecules [42, 43, 49] being an important improvement but without phenotype reversion. Just lately, genetic correction using CRISPR and TALEN was able to correct the dystrophin and permit normal gene expression of dystrophin [22].

Considering an acupuncture therapy for mdx and the results found here, acupuncture associated or not with SHED cells seems to increase dystrophin production and prevent the disease progression, being revealed as an alternative treatment for this untreated disease. An improvement in dystrophin expression was also observed in SHED/false acupoint suggesting the influence of cells in the treatment. Moreover, CPK decrease was observed in all treatments, but only SHED/true acupoint treatment was able to improve force, an important clinical parameter to DMD patients. In this work we can infer that acupuncture associated with SHED has a beneficial effect in muscle force as well as dystrophin expression and CPK decrease.

## 4. Conclusions

The use of acupuncture associated with SHED therapy appears to have a benefic effect in reducing disease progression in mdx mice and so far open new perspectives and opportunities to DMD treatment or could, at least, improve the quality of patient's life.

## Figures and Tables

**Table 1:** Wire test score and the strength quantification. This table reveals the score attributed for each action using wire test. The total time used in this evaluation was 21 seconds. The number of times walking or holding on the wire was attributed considering 0 to 4 steps. For each animal, the points obtained from Table 1 were summed and according to the score obtained the animal was classified in poor (0–2 points), median (3–5 points), and strong (6–8 points).

Action	0 step/holding until 9 s on the wire	1 to 3 steps/holding 10–20 s on the wire	4 or > turn/21 s
Raise the body above the wire	0 point	1 point	2 points
Walk on the wire	0 point	1 point	2 points
Put the pelvic member on the wire	0 point	1 point	2 points
Time on the wire (seconds only)	0 point	1 point	2 points
